# A Serious Game for Soft Skills Assessment in Human Resources: Cross-Sectional Within-Participant Convergent Validity Study

**DOI:** 10.2196/86054

**Published:** 2026-08-19

**Authors:** Maxime Boutrouille, Léo Fichet, Jérôme Dinet

**Affiliations:** 1 Yuzu, 2 Rue Maurice Barrès, Metz, 57000, France, 33 637401015; 2 Laboratoire de Psychologie et Neurosciences et Chaire industrielle "BEHAVIOUR", Campus Lettres et Sciences Humaines, Université de Lorraine, Nancy, France

**Keywords:** soft skills, assessment, serious game, video game, validity

## Abstract

**Background:**

Soft skills are increasingly assessed in human resources, but commonly used methods (eg, interviews and self-report questionnaires) have well-known limitations. Serious games have been proposed as a complementary assessment format because they can standardize administration, embed assessment in interactive scenarios, and capture behavioral traces. However, evidence for their psychometric validity remains limited and heterogeneous. Establishing convergent validity against well-established reference instruments is a key step in supporting their use as assessment tools.

**Objective:**

This study aimed to evaluate the convergent validity of Yuzu, a serious game that assesses (1) active listening via a gamified questionnaire inspired by the Active-Empathic Listening Scale (AELS), (2) decision-making under uncertainty via a gamified adaptation of the Iowa Gambling Task (IGT), and (3) teamwork style via dialogue choices inspired by the SYMLOG (System for the Multiple Level Observation of Groups) model.

**Methods:**

We conducted a cross-sectional, within-participant convergent validity study in France with 39 adults (n=23 women; mean age 27.79, SD 7.96 y). Participants completed a single laboratory session on a desktop PC with headphones (Yuzu build v2, developed by Yuzu). Participants completed the 3 Yuzu modules and the corresponding reference instruments, administered separately (the AELS, an online IGT via PsyToolkit, and a simplified SYMLOG questionnaire). Primary outcomes were the associations between Yuzu and reference scores for each construct (active listening total score, IGT exploitation-phase net score, and SYMLOG dimension scores). Convergent validity was examined using Spearman correlations (2-sided α=.05). Agreement was additionally examined using Bland-Altman analyses for active listening and equivalence testing using two one-sided tests (TOST) for IGT net scores.

**Results:**

At α=.05, active listening showed strong convergence between Yuzu and the AELS total score (Spearman ρ=0.890, 95% CI 0.804-0.948; *P*<.001), with minimal systematic bias (mean difference of 0.024, 95% CI −0.031 to 0.078). Decision-making scores were statistically equivalent across modalities based on TOST. The mean net score difference was 1.35 (90% CI −1.22 to 3.93), within the equivalence bounds [−5,+5] (TOST lower: *P*=.001; TOST upper: *P*=.01). Teamwork dialogue scores did not converge with the SYMLOG dimensions (dominance: ρ=−0.070, 95% CI −0.420 to 0.280; *P*=.69; positivity: ρ=0.082, 95% CI −0.266 to 0.418; *P*=.64; task orientation: ρ=0.134, 95% CI −0.260 to 0.494; *P*=.44), consistent with a ceiling effect toward cooperative choices.

**Conclusions:**

This study provides convergent validity evidence for 2 complementary assessment modalities embedded in a single serious game, showing that a gamified AELS-inspired module and a gamified IGT adaptation can closely match established reference measures while supporting standardized administration. In contrast, the dialogue-choice teamwork module showed limited sensitivity and no convergence, suggesting that interpersonal profiling in serious games may require more discriminating scenario design and stronger controls for social desirability. Unlike many previous studies that evaluated a single game component, this study provides a module-by-module convergent validity blueprint within a single platform by using matched reference instruments, thereby informing both research and human resources deployment.

## Introduction

Game-based assessments and serious games are increasingly used in human resources to evaluate job-relevant competencies, partly because they are expected to improve applicant reactions and limit response distortions while preserving measurement quality [1-4]. However, recent evidence syntheses converge on a more cautious conclusion: empirical support remains uneven, construct validity findings are often inconsistent, and any advantages over conventional methods appear smaller and more contingent on design choices than is sometimes implied in applied discourse [4]. This lack of robust and generalizable validity evidence is all the more concerning because these tools are frequently positioned as solutions for assessing soft skills.

Soft skills are widely treated as important determinants of employability and job performance in contemporary labor markets [5]. Technological change has shifted the comparative advantage of human work away from routine execution and toward capacities such as judgment, flexibility, and creativity [5,6]. In project-based and technology-driven settings, individuals must interpret dynamic social contexts, coordinate with others, and adapt their behavior under constraints, making these transversal competencies practically consequential for organizations [7,8]. Although there is no universal consensus on what should be labeled as “soft skills” [9,10], the diversity of terms used in the literature—including nontechnical skills [11], transversal skills [12], adaptive skills [13], and sociocognitive skills [13]—further illustrates the construct’s heterogeneity. Therefore, a working definition is needed to guide its operationalization and validation. In this study, we adopt the definition proposed by Haselberger et al [14], which is widely cited in this literature [15-17]: “Soft Skills represent a dynamic combination of cognitive and meta-cognitive skills, interpersonal, intellectual and practical skills.” From an assessment standpoint, this definition implies that soft skills should ideally be captured through indicators that are meaningful in context rather than only through what individuals report about themselves.

Several methods are routinely used to assess soft skills in applied settings, but each raises methodological limitations. Interviews and rater-based judgments can be informative; however, they remain sensitive to evaluator variability, bias, and indirect discrimination, and they do not always provide a standardized basis for comparison across candidates [18-21].

Self-report questionnaires are commonly used because they are practical and interpretable, and they can capture introspective information [22,23]. However, self-report measures are vulnerable to systematic response biases and method effects, including anchoring, primacy and recency effects, time pressure, and consistency motivation [22]. In selection contexts, socially desirable responding, acquiescent responding, and extreme responding are additional threats to validity [24]. As a result, questionnaires may capture self-perceptions or self-presentational strategies rather than performance in work-relevant situations.

Cognitive tests can also be used as indicators of abilities related to adaptive performance, for example, when cognitive flexibility supports adaptation or decision-making as a component of problem-solving [25,26]. For instance, progressive matrices proposed by Raven have long been used as a benchmark for reasoning [25]. However, applicant reactions to cognitive testing can be negative, including perceptions that tests are poorly related to the job. Cognitive testing also raises fairness concerns because it can yield substantial group differences and may induce test anxiety or stereotype threat, potentially undermining both performance and acceptability [27-31].

In response to these limitations, serious games have been proposed as a complementary assessment format because they can place candidates in interactive situations and enable observation of in situ behavior [1]. They can offer more immersive experiences [32,33], enhance perceived attractiveness [1-3], and provide access to behavioral traces, such as response sequences, decision trajectories, and reaction times [34,35]. Some experimental work further suggests that game-based contexts may mitigate certain social biases, for example, by reducing stereotype threat effects on performance [36].

However, the current evidence base indicates that these potential benefits do not translate uniformly into stronger measurement. Validity remains insufficiently documented for many tools [4,28]. More broadly, the validity of game-based assessments appears heterogeneous and dependent on the targeted construct and design features, which limits generalization across tools and contexts [4]. Additional challenges include face validity concerns when tasks appear disconnected from the claimed competency, construct contamination when multiple attributes are blended within a single activity, and reduced trust when scoring relies on opaque feature extraction or machine learning [28].

A practical implication is that validity evidence should be established module by module, with explicit reporting of how each construct is operationalized within gameplay and how closely the resulting scores align with well-established reference measures [4]. To address this need, we developed Yuzu, a serious game designed to assess soft skills through a multimodal framework. Building on prior feasibility work showing that classical psychometric paradigms can be adapted within Yuzu [37], the present study evaluates convergent validity across 3 modules representing distinct measurement modalities: (1) a gamified questionnaire module targeting active-empathic listening, inspired by the Active-Empathic Listening Scale (AELS) [38,39]; (2) a gamified decision-making task targeting decision-making under uncertainty through an adaptation of the Iowa Gambling Task (IGT) [40]; and (3) a dialogue-choice module targeting teamwork style based on constructs from the SYMLOG (System for the Multiple Level Observation of Groups) framework [41] and compared with scores from a simplified SYMLOG questionnaire [42].

We used a cross-sectional, within-participant convergent validity design in which the same participants completed each Yuzu module and its corresponding reference measure administered separately. We formulated one primary hypothesis regarding convergent validity across modules. Specifically, we hypothesized that (H1a) scores from Yuzu’s active-empathic listening module would be positively correlated with the AELS total score, that (H1b) performance in Yuzu’s decision-making module would be equivalent to performance observed in the reference IGT version, and that (H1c) the dimensions derived from Yuzu’s teamwork dialogue module would be positively correlated with the corresponding dimensions measured by the SYMLOG questionnaire.

## Methods

### Inclusion and Exclusion

A total of 39 participants completed the full laboratory session and were included in the analyses. Participants were eligible if they were aged 18 years or older and had sufficient French proficiency to understand the study instructions and complete all study materials. The study had no a priori exclusion criteria beyond these requirements. For each module-level analysis, participants were excluded only if paired data were incomplete for that module (ie, if either the Yuzu module score or the corresponding reference measure score was missing).

### Participant Characteristics

The sample comprised 39 participants, including 23 (59.0%) women and 16 (41.0%) men. The mean age was 27.79 (SD 7.96; range 20-54) years. Participants’ occupational status was as follows: students (n=16, 41.0%), executives or managers (n=14, 35.9%), employees (n=6, 15.4%), retired participants (n=1, 2.6%), entrepreneur (n=1, 2.6%), and job seeker (n=1, 2.6%). Self-reported computer literacy was high on average (mean 7.91, SD 2.11), and familiarity with video games was moderate-to-high (mean 6.67, SD 2.69; Table 1).

**Table 1.** Demographic characteristics of participants in the convergent validity study (N=39).

Variable	Values
Gender, n (%)	
Women	23 (59.0)
Men	16 (41.0)
Age (y), mean (SD), range	27.79 (7.96), 20‐54
Education and occupational status, n (%)	
Students	16 (41.0)
Executives or managers	14 (35.9)
Employees	6 (15.4)
Retired participants	1 (2.6)
Entrepreneur	1 (2.6)
Job seeker	1 (2.6)
Computer literacy, mean (SD)	7.91 (2.11)
Video game familiarity, mean (SD)	6.67 (2.69)

### Sampling Procedures

#### Recruitment Setting, Location, and Dates

Participants were recruited in France between November and December 2024 through a study advertisement posted on LinkedIn. Interested individuals contacted the research team and were scheduled for an in-person laboratory session. Data collection took place in controlled laboratory settings at the Centre for Research in Psychology: Cognition, Psychism, and Organizations (CRP-CPO, University of Picardie Jules Verne) and the Lorraine Laboratory of Psychology and Neuroscience of Behavioral Dynamics (2LPN, University of Lorraine). All sessions were conducted in quiet rooms without distractions to ensure standardized testing conditions.

#### Sampling Method and Self-Selection

The study used convenience sampling with self-selection. A public recruitment post was published on LinkedIn, inviting adults to participate in a laboratory-based study. Interested individuals self-referred by contacting the research team and were screened for eligibility based on the inclusion and exclusion criteria. Eligible individuals were then scheduled for an in-person laboratory session.

### Sample Size, Power, and Precision

#### Intended and Achieved Sample Size

The intended sample size was approximately 40 participants, primarily determined by feasibility constraints for in-laboratory testing and by the objective of detecting at least moderate convergent associations in an early-stage validation study. The achieved sample size was 39 participants for the full session. For the teamwork module, the analytic sample comprised 35 participants because game data for that module were not available for 4 participants.

#### Power and Precision Rationale

With a sample size of 39 participants, the study has approximately 80% power (2-sided α=.05) to detect correlations of *r* or ρ=0.44 or greater. For the teamwork module (n=35), the corresponding threshold was approximately 0.46. Therefore, the study was not designed to reliably detect small associations. No interim analyses or stopping rules were prespecified.

### Measures and Covariates

#### Primary Measures

This convergent validity study compared 3 assessment modalities embedded in the serious game Yuzu with corresponding reference instruments administered outside the game. Active-empathic listening was assessed in Yuzu using a questionnaire module based on the AELS [38,39]. The reference measure was the original AELS questionnaire [38,39]. Decision-making under uncertainty was assessed in Yuzu using an adaptation of the IGT [40]. The reference measure was the original IGT administered online [43]. Teamwork-related interpersonal behavior was assessed in Yuzu through structured dialogue choices, operationalized using the SYMLOG framework [41]. The reference measure was the simplified SYMLOG questionnaire by Blumberg [42].

#### Secondary Measures and Covariates

To evaluate whether performance could be biased by participants’ relationships with digital technology, measures of computer literacy and technophilia were collected. In addition, participants completed a game experience questionnaire based on the playability concept [44] and a demographic questionnaire during the debriefing phase. No other covariates were included in the primary inferential analyses.

### Data Collection

**Figure 1.**
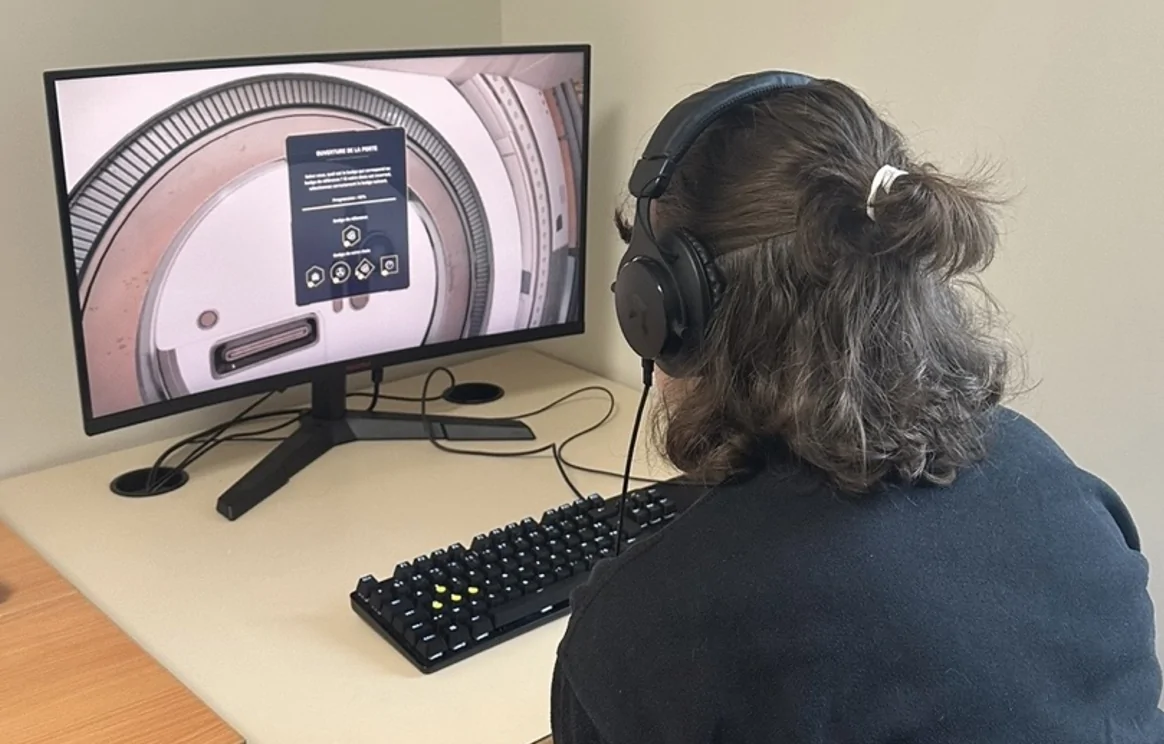
Experimental setup used during the laboratory session.

All participants completed a single laboratory session lasting approximately 2 hours, including a 10-minute break. Tasks were completed on a desktop PC with a curved monitor and over-ear headphones, using Yuzu build v2 developed by Yuzu (Figure 1).

To control for potential order effects, the administration was counterbalanced: half of the participants completed the Yuzu tasks first and then the reference tests, whereas the other half completed the reference tests first and then Yuzu. The session included (1) a welcome and briefing with an explanation of the objectives and the session outline; (2) completion of the Yuzu tasks and reference tests; and (3) a debriefing interview, a game experience questionnaire, and a demographic questionnaire.

### Quality of Measurements

Several procedures were used to enhance measurement quality and standardization. Data were collected in controlled laboratory environments using standardized hardware and with minimal distractions. Instructions and session timing were standardized across study sites. Outcome scoring for the Yuzu modules was automated through embedded trackers to minimize rater-dependent variability. Order effects were addressed through counterbalanced administration of Yuzu and the reference measures.

### Instrumentation

#### Serious Game Platform

**Figure 2.**
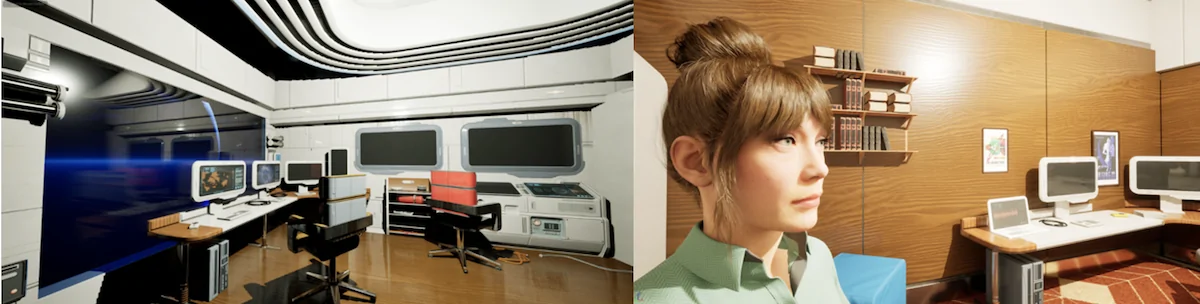
In-game screenshots from Yuzu illustrating the 3D environment used in the assessment tasks.

Yuzu is a single-player serious game for soft skills assessment that integrates multiple modalities within a single narrative 3D environment. Players navigate a space station, interact with nonplayable characters, complete tasks, and make decisions under constraints (Figure 2).

All actions are recorded through embedded trackers, producing behavioral indicators such as response patterns and choice sequences. Yuzu was designed by a multidisciplinary team (work psychology and ergonomics, engineering, and user interface design) in collaboration with the 2LPN research laboratory (University of Lorraine, France).

#### Module 1: Active-Empathic Listening (Questionnaire Modality)

**Figure 3.**
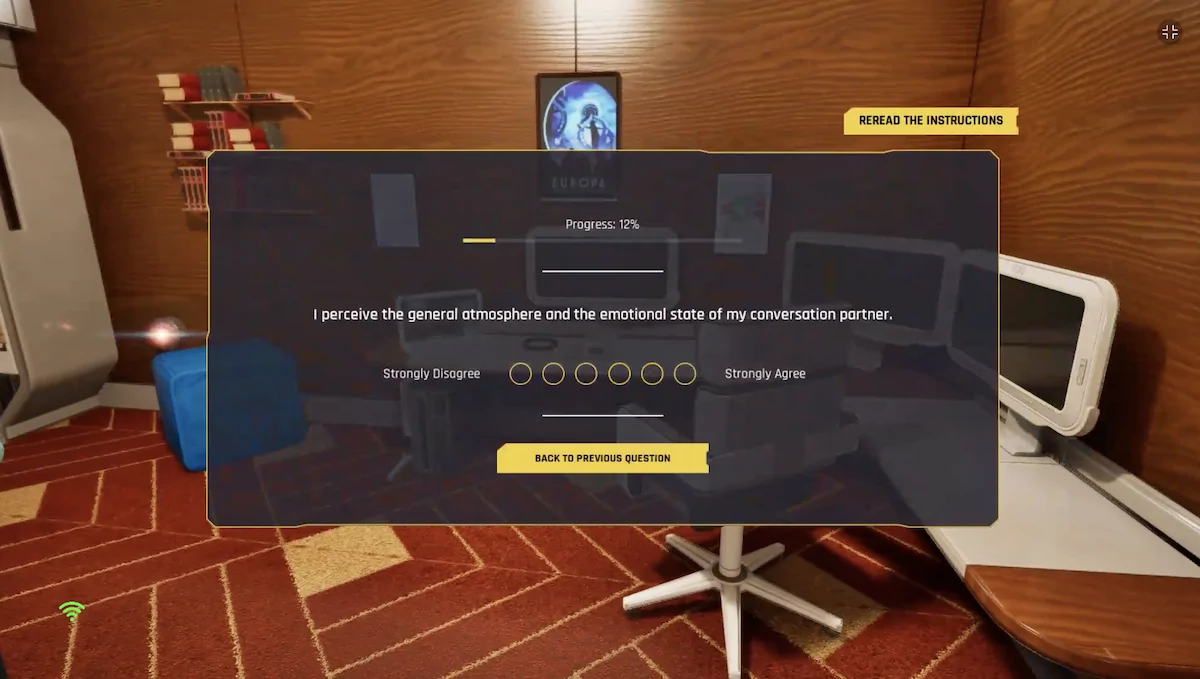
Screenshot of the Yuzu active listening module, inspired by the Active-Empathic Listening Scale.

Active listening is commonly assessed using self-report scales because it reflects cognitive and emotional processes that can be difficult to capture through performance tasks. The AELS is a widely used and validated tool for measuring listening quality in interpersonal communication [38,39]. It distinguishes 3 components: sensing, processing, and responding. In Yuzu, these components served as the conceptual framework for an original in-game questionnaire module. Items were specifically formulated for the serious game, and responses were collected using a 5-point Likert-type scale. The module yields an overall active listening score and 3 subscores corresponding to sensing, processing, and responding. These were compared with the reference AELS questionnaire [38]. The interface of the active listening module is shown in Figure 3.

Within the narrative, this module appears after the player awakens from a prolonged sleep: the station’s physician administers a short questionnaire to verify the participant’s condition and adaptation to the situation.

#### Module 2: Decision-Making Under Uncertainty (Task Modality)

We selected the IGT [40] as the reference paradigm for decision-making under uncertainty. In the classical IGT, participants complete 100 trials and choose among 4 decks (A, B, C, and D). Decks A and B are disadvantageous over the long term (high immediate gains but long-term losses), whereas decks C and D are advantageous (lower immediate gains but long-term benefits). Expected learning is reflected in an increasing preference for advantageous decks over the course of the task.

**Figure 4.**
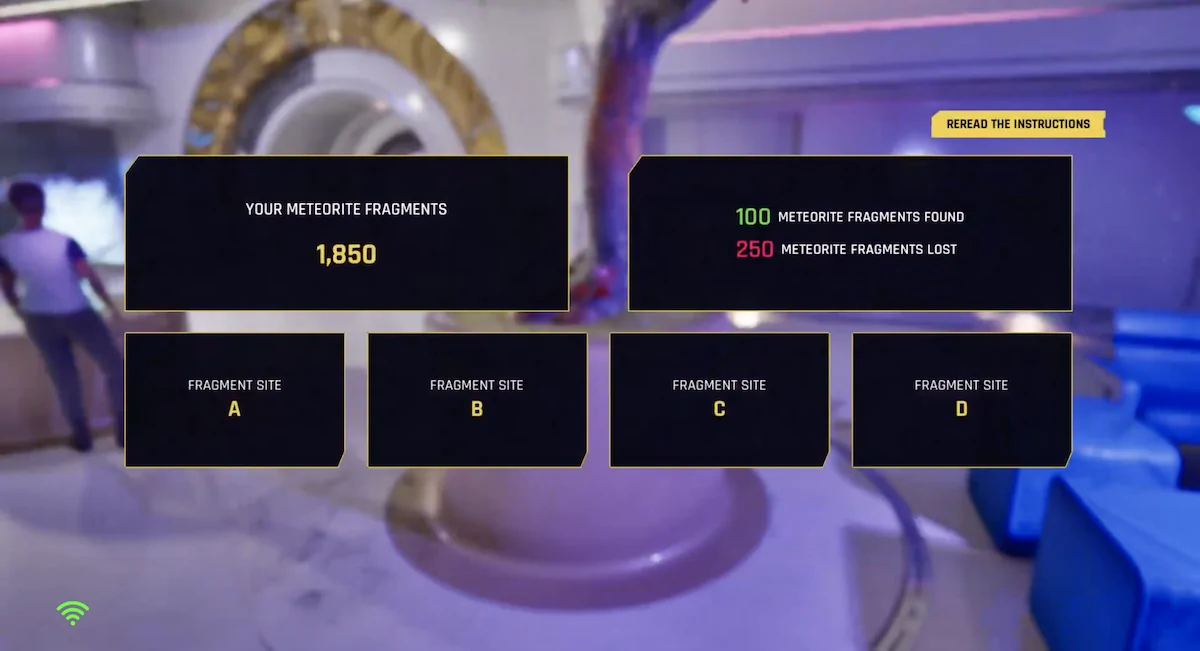
Screenshot of the Yuzu decision-making module (an adaptation of the Iowa Gambling Task).

In Yuzu, the core principles of the IGT were retained and transposed into a serious game format in which participants collected meteorite samples using drones across 4 excavation sites. The 100 trials were divided into 10 blocks of 10 choices each. Consistent with prior work [45], the first 40 trials (blocks 1-4) were treated as an exploration phase and the remaining 60 trials (blocks 5-10) as an exploitation phase. The interface of the game-based IGT adaptation is shown in Figure 4.

#### Module 3: Teamwork (Dialogue-Choice Modality)

Interpersonal behavior in team settings was operationalized using the SYMLOG framework [41], which positions actions along core social dimensions such as dominance, affiliation, and task orientation. The Yuzu teamwork scenario places the player in an emergency situation in which a spacecraft approaches the space station dangerously and rapid decisions must be made. Participants completed 4 sets of dialogue choices during interactions with nonplayer characters. Response options varied in both content and interpersonal tone (eg, more or less dominant or friendly), and some proposals originated from Earth as an external authority, allowing players to either follow or reject directives.

**Figure 5.**
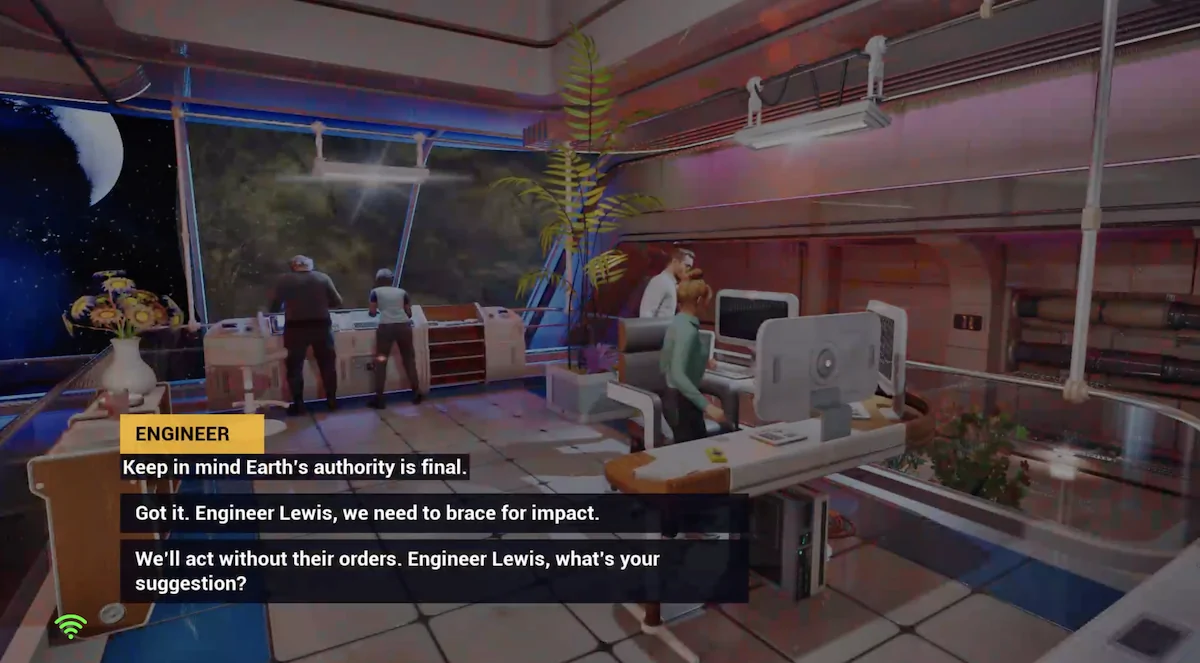
Screenshot of the Yuzu teamwork module (adaptation of the SYMLOG [System for the Multiple Level Observation of Groups] model).

Each response option was precategorized along 3 axes aligned with SYMLOG: dominance vs submission, friendliness vs hostility, and acceptance vs rejection of norms and tasks. For each participant, mean scores were computed for these dimensions to derive an in-game interpersonal profile, which was compared with the simplified SYMLOG questionnaire [42] administered outside the game. The dialogue-choice interface used in the teamwork module is shown in Figure 5.

### Masking

No masking procedures were implemented. Participants were aware that they were completing both the serious game modules and the corresponding reference measures within the same session. Because primary outcomes were computed algorithmically from response data and in-game trackers, there were no subjective raters whose condition assignment would require masking. Data were analyzed using deidentified datasets.

### Psychometrics

The reference tests (AELS [38,39], IGT [40], and simplified SYMLOG [42]) have published validation evidence. In the present study, convergent validity was examined by correlating the in-game module scores with scores from these reference questionnaires.

### Conditions and Design

This study used a quantitative, cross-sectional, within-participant convergent validity design. No experimental manipulation of conditions was implemented; therefore, the design is best categorized as a nonexperimental observational design. Each participant completed, within the same session, each Yuzu module and its corresponding reference measure (in a counterbalanced order). This study is reported in accordance with the APA (American Psychological Association) *JARS-Quant* (*Journal Article Reporting Standards for Quantitative Research*) guidelines [46].

### Data Diagnostics

Analyses were conducted on complete paired observations for each module, meaning that participants contributed to an analysis only when both the Yuzu score and the corresponding reference score were available. For the teamwork module, paired game data were unavailable for 4 participants; thus, the teamwork analyses were conducted with 35 participants. Because the primary correlational analyses were nonparametric (Spearman ρ), these analyses are less sensitive to distributional nonnormality and outliers than Pearson correlations. Data distributions and descriptive statistics were inspected prior to inferential analyses. No data imputation was performed.

### Analytic Strategy

All tests used a 2-sided α level of .05. The primary hypothesis (H1) was that each Yuzu module would demonstrate convergent validity with its corresponding reference measure. For the active-empathic listening module, convergent validity was evaluated using Spearman rank correlations between the Yuzu scores and the AELS reference scores. CIs for the correlations were estimated through bootstrap resampling (bias-corrected and accelerated [BCa], 5000 resamples). Absolute agreement was additionally evaluated using Bland-Altman analysis [47] (mean bias and 95% limits of agreement). Relative agreement was quantified using the Lin concordance correlation coefficient, computed using the SimplyAgree module in Jamovi [48].

For the decision-making module, IGT performance was summarized using the net score in the exploitation phase (blocks 5-10), defined as (C + D) − (A + B). Equivalence between the Yuzu version and the reference IGT was tested using the two one-sided tests (TOST) procedure [49], with an a priori equivalence margin of ±5 points for the net score. Block-wise descriptive analyses were conducted to visualize learning trajectories across the task.

For the teamwork module, each in-game dialogue choice was mapped to the 3 SYMLOG dimensions, and mean scores were computed per participant. Convergent validity was evaluated using Spearman rank correlations between the Yuzu-derived dimension scores and simplified SYMLOG questionnaire scores [42], with 95% bootstrap CIs (BCa, 5000 resamples). Given the small number of theory-driven module-level comparisons aligned with prespecified hypotheses, *P* values were interpreted alongside effect sizes and CIs. Exploratory analyses included block-wise learning curves for the IGT and inspection of agreement patterns in the active listening module using Bland-Altman plots.

### Ethical Considerations

This study was conducted in accordance with French regulations governing noninterventional behavioral research (Loi Jardé, L1121-1-1). The protocol involved no medical procedures, no clinical intervention, and no collection of health-related or sensitive personal data. Under applicable French regulations, submission to a Comité de Protection des Personnes (CPP) or formal institutional review board (IRB) approval was not required. Consequently, no IRB approval number was assigned. All participants provided written informed consent prior to participation. The consent form specified that participation was voluntary, that withdrawal was possible at any time without justification, and that the data would be used exclusively for research purposes. Research data were deidentified prior to analysis and stored on password-protected servers accessible only to the research team. Participants did not receive monetary compensation. As an acknowledgment of their time, they received a detailed feedback report based on their soft skills. The research participant appearing in Figure 1 provided written informed consent for publication of the photograph.

## Results

### Participant Flow

**Figure 6.**
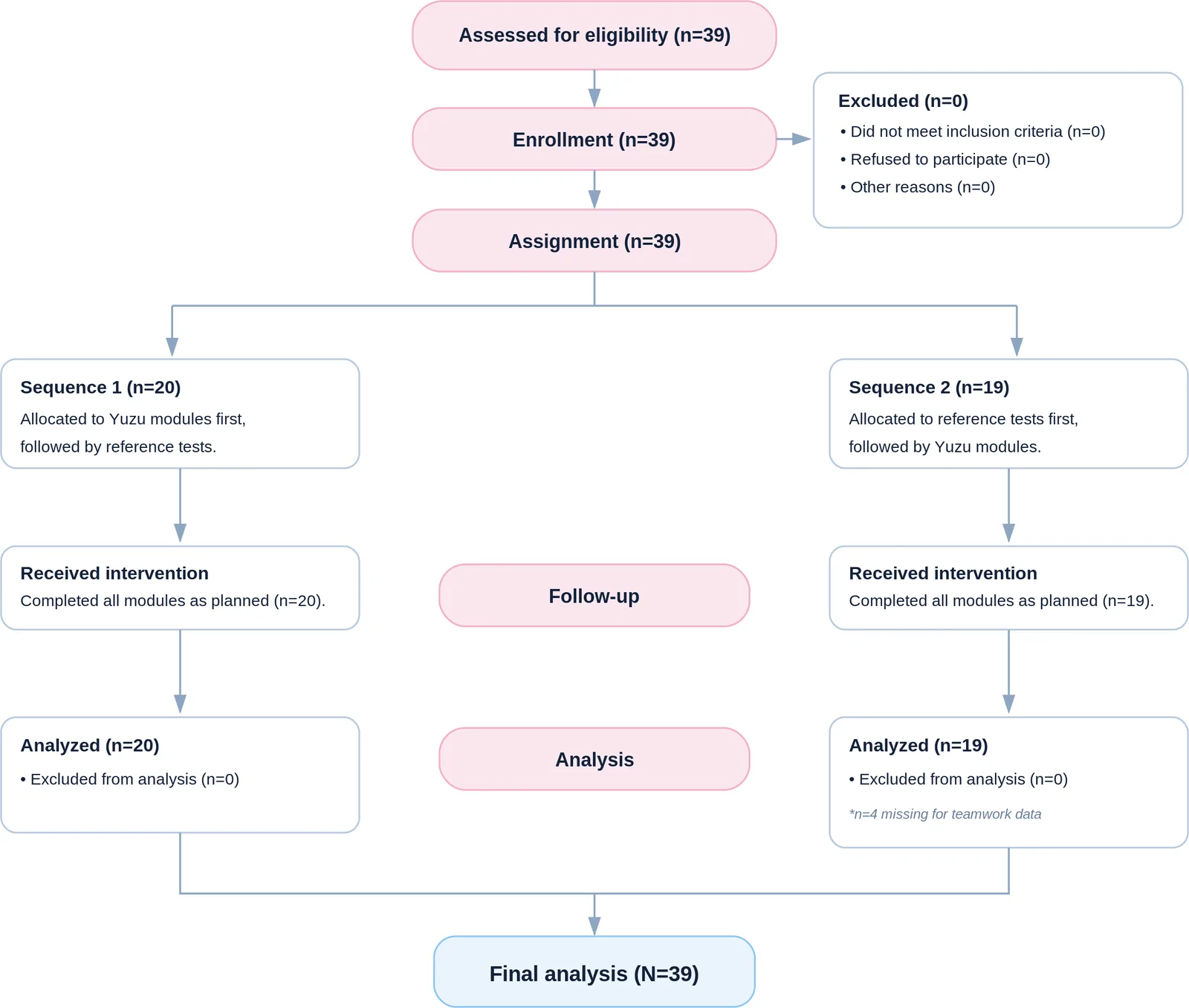
Flowchart of the experimental procedure and assessment sequence.

A total of 39 participants were assessed for eligibility, enrolled, and completed the laboratory session. Complete paired data were available for 39 participants in the active-empathic listening and decision-making modules. For the teamwork module, complete paired data were available for 35 participants because the in-game teamwork data were not saved for 4 participants due to a technical issue. Figure 6 presents the participant flow.

### Recruitment

Recruitment and data collection were conducted in France between November 2024 and December 2024. This study used a single-session, cross-sectional design with no follow-up assessments.

### Statistics and Data Analysis

#### Analysis Strategy

All statistical tests used a 2-sided α level of .05. For equivalence testing, the TOST procedure was conducted at α=.05; therefore, 90% CIs are reported for equivalence tests, whereas 95% CIs are reported elsewhere. Missing data were limited to the teamwork module: 4 of 39 participants (10.3%) had missing in-game teamwork data because the module data were not saved. These cases were excluded from teamwork analyses only, resulting in an analytic sample size of 35 participants. No imputation was performed. Because the missingness was caused by a technical data-saving issue, it was assumed to be unrelated to the underlying teamwork construct; however, this assumption cannot be formally verified.

Results are reported in the order of the hypotheses stated in the *Introduction* section. Listening and teamwork hypotheses were tested using Spearman rank correlations with bootstrap CIs. Decision-making equivalence was tested using TOST and complemented by a paired 2-tailed *t* test and an effect size estimate.

#### Active Empathic Listening Module (H1a)

Convergent validity was supported for active listening. Yuzu’s active listening score showed a strong association with the reference AELS total score (Spearman ρ=0.890, 95% CI 0.804-0.948; *P*<.001; 95% CIs estimated using bootstrap resampling [BCa, 5000 resamples]).

**Figure 7.**
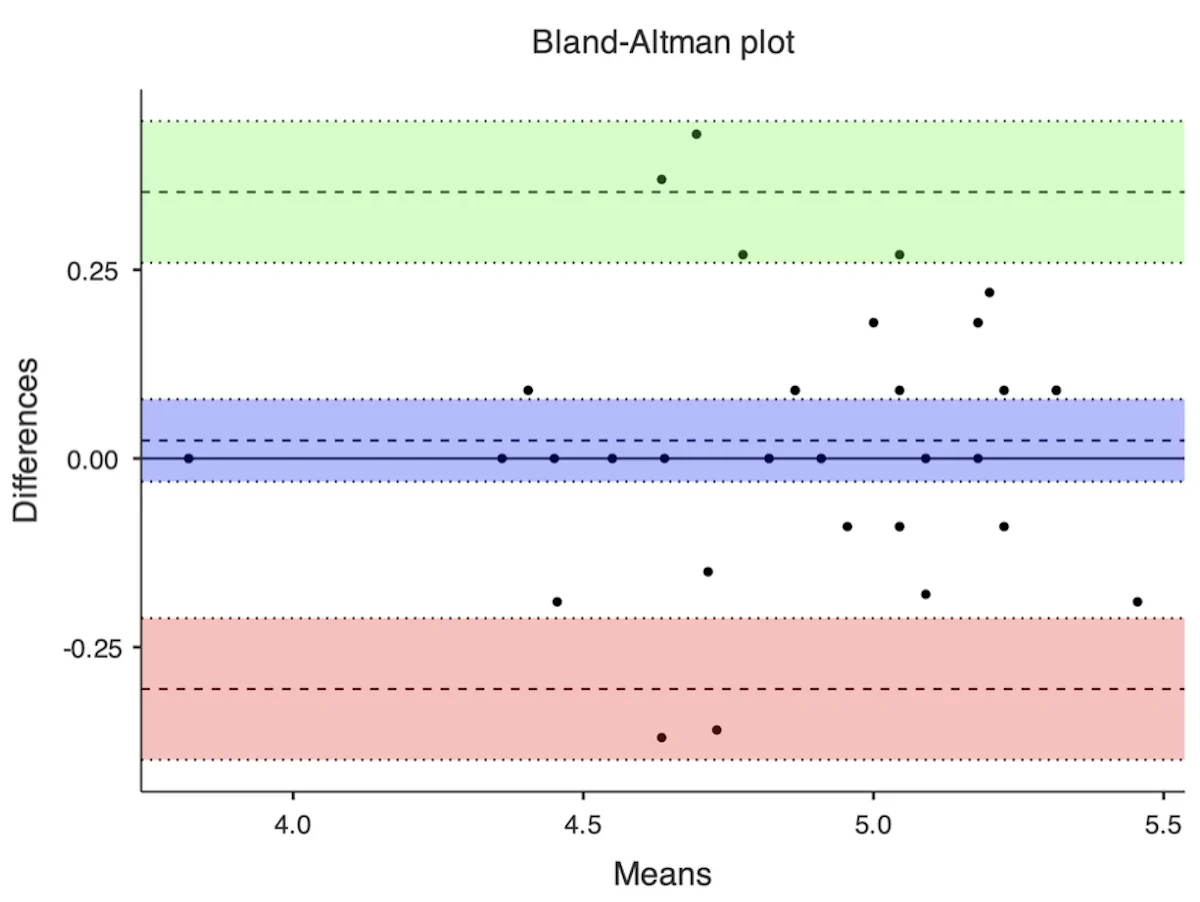
Bland-Altman plot between Yuzu’s active listening module and the reference Active-Empathic Listening Scale, suggesting the absence of systematic differences (N=39).

Agreement between the 2 versions was evaluated using Bland-Altman analysis (Table 2; Figure 7). The mean bias was close to 0, and the limits of agreement indicated no large systematic disagreement between the formats.

**Table 2.** Bland-Altman analysis between Yuzu’s active listening module and the reference Active-Empathic Listening Scale (N=39).

Agreement	Estimate (95% CI)
Bias (N=39)	0.0238 (–0.0306 to 0.0783)
Lower limit of agreement	–0.3056 (–0.3995 to –0.2117)
Upper limit of agreement	0.3533 (0.2594 to 0.4472)

The Lin concordance correlation coefficient (CCC) indicated good-to-excellent agreement between the 2 versions (CCC=0.899, 95% CI 0.817-0.946). Overall, these results support convergent validity and close agreement between Yuzu’s listening module and the reference AELS.

#### Decision-Making Module (H1b)

**Figure 8.**
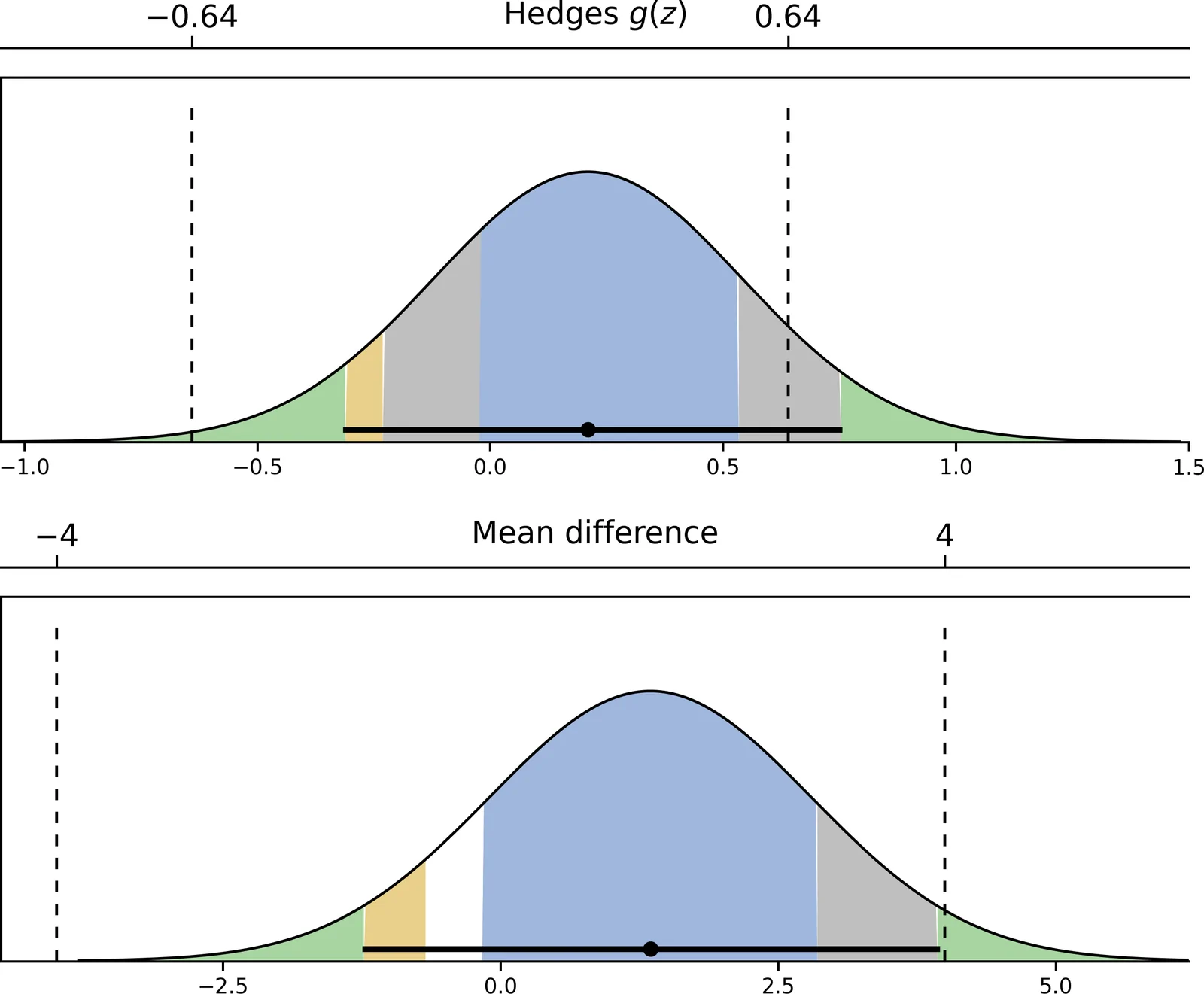
Two one-sided tests for equivalence between Yuzu and the reference Iowa Gambling Task. Net score: the 90% CI for the mean difference lies entirely within the equivalence bounds (±5), supporting equivalence (N=39).

The analyses supported the equivalence between the Yuzu adaptation and the reference IGT during the exploitation phase. The TOST 90% CI for the mean difference fell entirely within the prespecified equivalence interval of −5 to +5, and both one-sided tests were significant (Table 3; Figure 8). The paired 2-tailed *t* test was not statistically significant, and the standardized effect size was small (Table 3).

**Table 3.** Two one-sided tests (TOST) equivalence and paired *t* tests comparing the reference Iowa Gambling Task with Yuzu’s game-based version, confirming equivalence (N=39).

Statistic	Estimate	*P* value	Equivalence bounds (low, high)
*t* test (net score)	0.913	.37	—^a^
TOST lower	3.615	.001	—
TOST upper	–1.789	.01	—
Effect size (raw; 90% CI)	1.352 (–1.224 to 3.927)	—	–5.000, 5.000
Effect size (Hedges *g*[*z*]; 90% CI)	0.210 (–0.311 to 0.752)	—	–0.637, 0.637

a Not applicable.

**Figure 9.**
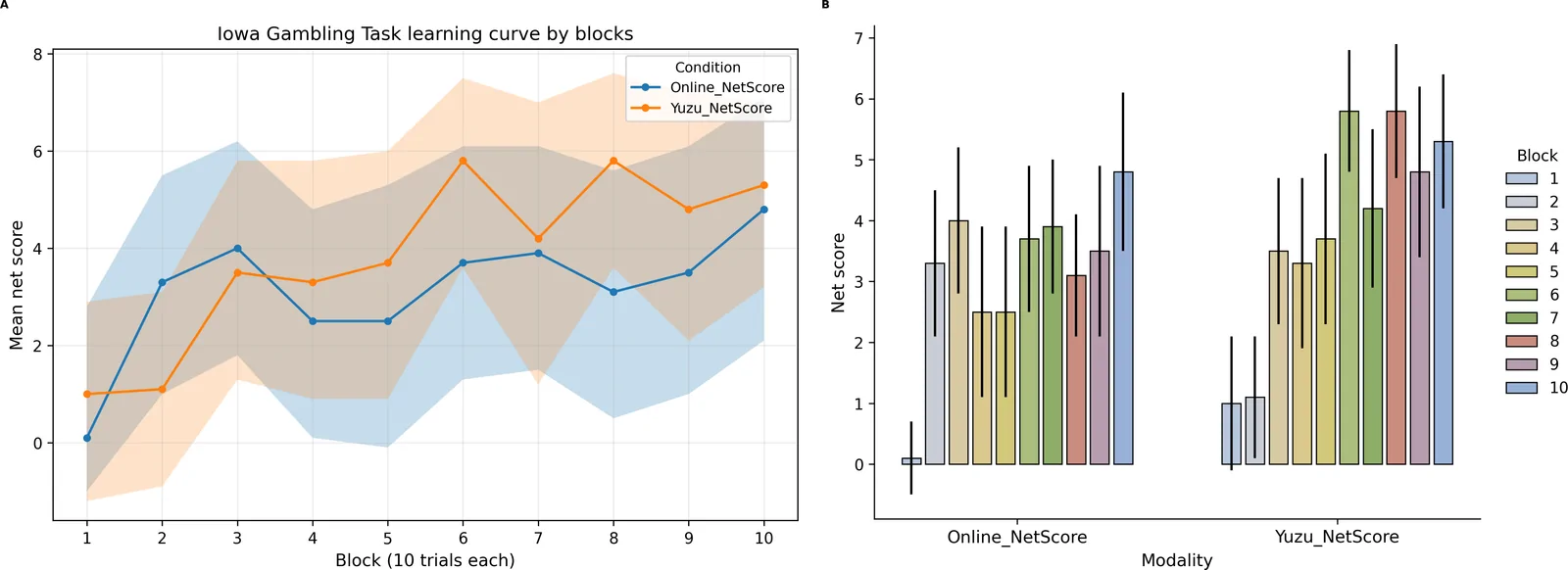
Evolution of the mean Iowa Gambling Task net score by 10-trial blocks for Yuzu and the reference task. (A) Mean net score across blocks for both modalities. (B) Mean net score by block and modality. Both conditions show a similar upward learning trajectory from exploration (blocks 1‐4) to exploitation (blocks 5‐10; N=39).

To describe learning dynamics, Figure 9 shows the evolution of the mean net score across blocks of 10 trials for both modalities. In both conditions, performance increased across blocks, with a similar shift from exploration to exploitation beginning at block 5.

#### Teamwork Module (H1c)

Convergent validity was not supported for the teamwork module. Yuzu-derived teamwork profiles were clustered toward a highly cooperative style, consistent with a ceiling effect. Spearman rank correlations between Yuzu’s 3 dimensions and the corresponding questionnaire dimensions were not statistically significant (Table 4).

**Table 4.** Spearman correlations between SYMLOG (System for the Multiple Level Observation of Groups) dimensions derived from Yuzu’s teamwork module and the reference SYMLOG questionnaire, rejecting convergence (n=35)^a^.

Dimension	Variable	Spearman ρ (95% CI)	*P* value
Dominance	Reference vs Yuzu	–0.070 (–0.420 to 0.280)	.69
Positivity	Reference vs Yuzu	0.082 (–0.266 to 0.418)	.64
Task	Reference vs Yuzu	0.134 (–0.260 to 0.494)	.44

a 95% CIs for Spearman correlations were estimated using bootstrap resampling (BCa, 5000 resamples; n=35).

Descriptive distributions suggested that the questionnaire differentiated participants across the 3 axes, whereas the Yuzu-derived scores were compressed and shifted in a way consistent with limited variance (Table 5).

**Table 5.** Descriptive statistics for SYMLOG (System for the Multiple Level Observation of Groups) dimensions in the questionnaire vs the Yuzu dialogue module (n=35).

Dimensions	Median (minimum to maximum)
Paper_dominance—*z* score	–0.0778 (–2.01 to 2.128)
Yuzu_dominance—*z* score	–0.2395 (–1.70 to 1.948)
Paper_positivity—*z* score	–0.1340 (–2.22 to 1.608)
Yuzu_positivity—*z* score	0.5657 (–2.45 to 0.996)
Paper_task—*z* score	–0.1764 (–2.71 to 2.359)
Yuzu_task—*z* score	0.4219 (–2.29 to 1.025)

## Discussion

### Principal Findings

This study examined the convergent validity of 3 soft skill assessment modalities embedded in the serious game Yuzu by comparing each module with an established reference measure. Regarding the primary hypothesis (H1), the results provided partial support. Convergent validity was strongly supported for the active-empathic listening module (H1a), with a high correlation and close agreement between Yuzu and the AELS reference instrument, and for the decision-making module (H1b), with statistically equivalent results across the gamified and reference versions of the IGT. By contrast, convergent validity was not supported for the teamwork module (H1c), where in-game scores showed restricted variance and no meaningful association with the reference SYMLOG questionnaire. Taken together, these findings suggest that the validity of serious game modules is construct-specific and modality-specific, and cannot be assumed to generalize across the components of a single platform.

### Interpretation

#### Construct Operationalization and Assessment Modality

Taken together, these results suggest that serious games can support convergent validity for some soft-skill constructs and modalities, but that validity evidence is highly dependent on how the construct is operationalized in gameplay and on the extent to which the measurement modality introduces method-related variance [50,51]. In practice, modules that closely preserve the structure of a reference instrument (eg, questionnaire-based formats) or a well-defined task paradigm (eg, feedback-based decision tasks) may translate more readily into game-based formats, whereas dialogue-choice approaches to interpersonal profiling may require stronger differentiation in scenario design and response options to generate sufficient variance for construct validation.

#### Active-Empathic Listening

The very high correlation and strong agreement between Yuzu and the AELS indicate that the in-game questionnaire module produced scores that were closely aligned with the reference instrument. One interpretation is that the narrative embedding of the questionnaire did not distort the construct being measured while preserving the conceptual structure of the original scale [38,39]. However, because both measures rely on self-reported judgments, shared method variance is a plausible contributor to the observed strength of the association [51]. In addition, response biases associated with self-report, including impression management and socially desirable responding, may remain present even when the questionnaire is integrated into a game narrative [24]. Therefore, the present findings support convergent validity with an established self-report instrument, but they do not yet demonstrate that Yuzu captures listening behavior as it unfolds in real interactions.

#### Decision-Making

Equivalence results indicate that the Yuzu adaptation preserved the decision-making tendencies captured by the reference IGT during the exploitation phase. This finding suggests that the gamified interface and narrative context did not substantially alter the balance between immediate rewards and long-term outcomes that the task is intended to probe [40]. The parallel block-wise trajectories are consistent with the interpretation that participants learned from feedback in a comparable manner across both modalities, in line with previous work [45].

This result is noteworthy because it suggests that the structural properties of a well-defined cognitive paradigm can be preserved when transposed into a game narrative, provided that the core contingency structure, here the asymmetric reward schedule across decks, remains intact. From a measurement standpoint, task-based modules of this kind may be more robust to gamification than questionnaire or dialogue-based formats, because scoring relies on behavioral sequences rather than self-report, thereby reducing susceptibility to socially desirable responding [24]. Future work should examine whether this equivalence holds across different gamified implementations and population subgroups.

#### Teamwork

The absence of convergent validity for the teamwork module can plausibly be explained by restricted variance and a ceiling effect in the in-game indicators. If most participants select options that are clearly cooperative and norm-conforming, the resulting scores cannot differentiate interpersonal styles and will correlate poorly with questionnaire measures that capture stable individual differences. This aligns with the general expectation that restriction of range attenuates correlations [52]. This pattern may also reflect the scenario’s incentive structure. Cooperation might have been framed as the most sensible option for narrative success, making alternative responses appear irrational or socially inappropriate. Such tendencies are compatible with the broader literature on socially desirable responding, in which respondents may favor options that maintain a positive self-image or align with perceived expectations [24].

Another possibility is a construct mismatch. The simplified SYMLOG questionnaire assesses broad interpersonal tendencies, whereas the Yuzu dialogue choices may have captured context-specific decision strategies under an emergency narrative. Like situational judgment tests, the dialogue-choice module may primarily capture respondents’ implicit trait policies or knowledge about which interpersonal behaviors are effective in a given situation, whereas the SYMLOG questionnaire targets broader dispositional teamwork tendencies [53]. In that case, the lack of convergence could indicate that the game module measured situational behavior shaped by the scenario rather than the trait-like interpersonal profile assessed by the questionnaire.

From a measurement perspective, the current dialogue-choice format may also suffer from limited behavioral sampling. With only a small number of choice points, measurement precision is limited, and individual scores become sensitive to idiosyncratic responses to specific prompts. This issue is consistent with discussions of scenario-based assessment formats, where item sampling and option design strongly influence reliability and construct representation [4,54]. Increasing the number of observations, diversifying situations, and designing options that are less transparently “good” or “bad” could improve score reliability and increase variance.

### Implications

These results suggest that validity evidence for serious game assessment should be developed module by module, with careful attention to modality and construct operationalization. For constructs assessed via in-game questionnaires, embedding within a narrative may preserve measurement properties while potentially improving acceptability. However, future work should include multimethod validation that goes beyond self-report and explicitly addresses method variance [51].

For task-based modules, the decision-making findings support the feasibility of adapting classical paradigms into serious game formats while retaining key psychometric properties. Future research should evaluate the robustness across different devices and contexts of administration, test whether equivalence holds across subgroups, and further examine the psychometric characteristics of task-derived metrics [45]. For dialogue-based interpersonal assessment, the present findings indicate that simple “choice point” implementations may be vulnerable to ceiling effects and restricted variance when scenarios strongly favor cooperation or make socially desirable options transparent [24,52]. Future iterations should incorporate design strategies to increase behavioral differentiation, for example, by increasing the number of observations, diversifying contexts and interaction partners, introducing credible trade-offs, and reducing the transparency of one clearly superior option. Scenario-based assessment work suggests that option design and sampling of situations are central to capturing construct-relevant variability [54].

From an applied perspective, the present study provides preliminary evidence that a serious game can yield convergent validity for some soft-skill indicators (active listening and decision-making) while highlighting that other constructs (teamwork) require further design and validation work. Organizations considering game-based assessment should treat different modules as distinct measurement instruments and require clear validity evidence for each targeted competency.

In the longer term, serious games could complement existing assessment batteries by providing standardized behavioral data in interactive contexts and potentially improving acceptability. Candidate reactions to assessment procedures are known to influence perceived fairness and organizational attractiveness. Therefore, it is important to evaluate user perceptions alongside psychometric performance [2,27].

### Limitations and Suggestions for Future Research

Several limitations should be acknowledged. First, the relatively small sample size limits the statistical power of the analyses [55,56]. The relatively homogeneous sample also limits external validity, and replication in more diverse occupational groups is needed [57]. It is therefore possible that effects of small magnitude were not detected. A promising avenue for future research would be to increase sample sizes in subsequent empirical studies. Another point concerns the duration of the experimental sessions, which was relatively long in this study. Excessive session length may generate fatigue, reduce participants’ concentration, and affect engagement. This can particularly influence the quality of responses to questionnaires or the consistency of performance in game-based tasks [58]. Future studies could explore shorter and more modular formats, for example, by splitting the session into multiple parts or by offering abbreviated versions of the tests.

Finally, the limitations observed in the dialogue-choice assessment call for methodological improvements. The low level of correlation with the SYMLOG questionnaire may be explained by a lack of nuance in the response options, a social desirability bias (participants’ tendency to choose the option perceived as the most socially acceptable), or the fact that in-game dialogues and questionnaires may capture only partially overlapping facets of interpersonal communication [59,60]. Future studies could diversify scenarios, introduce more contrasting dilemmas, analyze not only the final choice but also response times and hesitation [61], and use AI-driven nonplayer characters to increase the credibility and sensitivity of the assessment.

### Conclusions

This study provides convergent validity evidence for 2 complementary assessment modalities embedded in a single serious game. Specifically, a gamified AELS-inspired module and a gamified IGT adaptation closely aligned with their corresponding reference measures under standardized administration, whereas the dialogue-choice teamwork module showed limited sensitivity and no convergence with questionnaire dimensions. More broadly, these findings support a modular approach to serious-game validation in human resources. Each module should be treated as a distinct measurement instrument requiring targeted validity evidence. The present work offers a concrete, replicable blueprint for module-by-module validation using matched reference measures and complementary analytic methods, and it highlights design requirements for interpersonal assessment (eg, reducing social desirability cues and increasing behavioral differentiation). Future research should extend this evidence base to predictive validity, subgroup robustness, and more ecologically rich social-interaction mechanics.

## Abbreviations

2LPN: Lorraine Laboratory of Psychology and Neuroscience of Behavioral Dynamics
AELS: Active-Empathic Listening Scale
BCa: bias-corrected and accelerated
CCC: concordance correlation coefficient
CPP: Comité de Protection des Personnes
CRP-CPO: Centre for Research in Psychology: Cognition, Psychism, and Organizations
IGT: Iowa Gambling Task
IRB: institutional review board
SYMLOG: System for the Multiple Level Observation of Groups
TOST: two one-sided tests
